# The feasibility and effectiveness of internet-based rehabilitation for patients with knee osteoarthritis: A study protocol of randomized controlled trial in the community setting

**DOI:** 10.1097/MD.0000000000022961

**Published:** 2020-10-30

**Authors:** Su-Hang Xie, Qian Wang, Li-Qiong Wang, Si-Yi Zhu, Yi Li, Cheng-Qi He

**Affiliations:** a School of Rehabilitation Sciences, West China School of Medicine; b Department of Rehabilitation Medicine Center, West China Hospital, Sichuan University; c Key Laboratory of Rehabilitation Medicine in Sichuan Province, West China Hospital, Sichuan University, Chengdu, Sichuan, P. R. China.

**Keywords:** community setting, internet-based rehabilitation, knee osteoarthritis

## Abstract

**Background:**

Internet-based rehabilitation can ease the progression of chronic diseases. There had been studies on internet-based rehabilitation of knee osteoarthritis (OA), but them were conducted at home and ignored the potential benefits in the community setting. This study will explore the feasibility and effectiveness of internet-based rehabilitation accompanies wearable devices in the community setting for the patients with knee OA.

**Methods:**

An assessor-blinded randomized controlled feasibility trial will be performed to study the feasibility and effectiveness of internet-based rehabilitation program for the patients with knee OA in the community setting. Forty participants with knee OA will be recruited and randomly allocated into internet-based rehabilitation group (IRG) or control group (CG). The interventions of IRG will be performed in the community setting via web-based platform and wearable devices. The outcome measures will be conducted at baseline, and post-intervention 6 weeks, 12 weeks during the study. The feasibility will be tested by the proportion of participants who will complete the internet-based rehabilitation program within 12 weeks in IRG as well as the compliance and satisfaction. Additionally, the effectiveness of internet-based rehabilitation will be assessed by the Western Ontario and McMaster Universities Osteoarthritis Index, 11-point Numerical Rating Scale and Short Form-36 quality-of-life questionnaire.

**Conclusion:**

The internet-based rehabilitation program and community-based interventions is feasible and efficacy to ameliorate the osteoarthritic pain and promote the physical function and quality of life for the patients with knee OA in the community setting.

**Trial registration number:**

The study was registered in the clinical trial registry ChiCTR2000033397.

## 1 Introduction

Osteoarthritis (OA) is a chronic, debilitating and degenerative joint disease, and widely considered as a significant threat to healthy aging.^[1]^ It has been reported that nearly 250 million individuals suffered from OA in the world, of which the prevalence of knee OA is the highest, accounting for 16–17% in the population aged from 50 to 75 years.^[2]^ The chronic pain and physiological dysfunction are considered to be the main problems of the patients with knee OA.^[1,3]^ To address these issues and control the progression of OA, lifestyle modification is one of the effective strategies universally recommended in the guidelines of OA. The lifestyle modification includes the physical fitting exercise, self-management of behavior, and efficient learning of osteoarthritis-related knowledge.^[4–6]^ Physical fitting exercise, such as aerobic exercise and strength training, plays an important role in balancing cytokine dynamics in the synovial fluid and inhibiting inflammatory reaction and oxidative stress.^[7,8]^ Self-management is one of the biopsychosocial models to promote the adherence to OA treatment through behavior change and psychosocial coping skills, which include the behavioral, role and emotional managements.^[9,10]^ The education of osteoarthritis-related knowledge is necessity for patients subjected to knee OA, which have been demonstrated to improve the quality of life and coping skills.^[11,12]^

It is a challenge to perform the lifestyle modification strategy to individuals with knee OA when they are discharged from health care setting.^[13,14]^ The lack of professional supervision and feedback would result in the decline of participation in continuous medical or rehabilitative services of OA, which will subsequently decrease the efficacy of OA treatments at home or in the community setting.^[15,16]^ Moreover, few medical facilities and resources could be accessible in the rural areas, where the elder had been reported to have poor health-related quality of life.^[17]^

With the development of telemedicine, patients living in remote areas could obtain a chance to perform a real-time communication with the professional physicians. The concept of telerehabilitation in the field of physical medicine and rehabilitation has been put forward, which combines telemedicine with rehabilitation interventions to provide support for the continuous rehabilitative services for the disabled patients.^[18]^ Of which, the internet-based rehabilitation is one of effective strategies in telerehabilitation, and its feasibility and effectiveness have been explored.^[19,20]^ The combination of an internet-supported program of constraint-induced therapy (LifeCIT) and a wearable real-time muscle kinematics device has been tested to improve the upper limb function and quality of life for people after stroke.^[19]^ Furthermore, internet-based rehabilitation was found to effectively reduce hospital admissions according to the summary of 47 eHealth applications used in Dutch COPD care.^[20]^ Internet-based rehabilitation had also been applied in the monitor, guidance and treatment of Parkinson's disease, post-knee arthroplasty and multiple sclerosis.^[21–23]^

Several studies have been conducted to explore the feasibility and effectiveness of the internet-based rehabilitation for individuals with knee OA, including the “Join2Move”, “PainCOACH” and “Help My Knees” programs.^[24–26]^ The compliance of internet-based rehabilitation varied in the previous studies, and appeared to be dependent on the education level and willingness of participants, as well as whether the remote-control reminder such as email, telephone or messages was set up during the study.^[25]^ It is therefore necessary to explore the feasible and effective way to improve the acceptability and adherence of internet-based rehabilitation for the patients with knee OA. Whilst the comparison of function and pain-relief was not significant between the internet-based rehabilitation and control groups, the programs based on the progressive lower-limb strength training, flexibility and walking exercise were shown to improve the function and ameliorate the osteoarthritic pain.^[26–28]^ The optimal design of modules with regard to the lifestyle modification in the internet-based rehabilitation programs should be taken into account for the patients with knee OA.

Community-based rehabilitation is a continuous rehabilitation model for patients with knee OA and it is also an important link from family to society.^[29]^ The World Health Organization had asserted the importance of enhancing the participation of people with disabilities in the daily life and social activities based on the International Classification of Functioning, Disability and Health framework.^[30]^ Participation is regarded as a vital outcome in community-based rehabilitation.^[31]^ Patients can reduce the sense of shame caused by diseases and build up confidence in a recognized or accepted environment, so as to promote the healthy development of physiology and psychology.^[32]^ It is also recognized that the development of community-based rehabilitation could facilitate the management of chronic diseases, in particular for OA.^[33]^ However, the insufficient number of healthcare providers in the community setting impeded the development of community-based rehabilitation for patients with OA.^[33,34]^ Researches showed that community-based rehabilitation could create a mutual supervision environment for patients and improve their compliance.^[35,36]^ In addition, wearable devices also promote the monitor of patients function and advance the community-based rehabilitative service, conveniently collecting data and giving feedback to clinicians.^[37,38]^ Up to date, there have been few studies published in China regarding the effect of internet-based rehabilitation on the continuous intervention for the patients with OA in the community setting. It is desirable to explore the optimal strategy of internet-based rehabilitation programs to improve the pain and physical function of the individuals with OA through the coordination of community-based rehabilitation in this study.

Therefore, the purpose of this randomized controlled trial is to explore the feasibility and effectiveness of combining the internet-based rehabilitation with the community-based interventions and resources for the patients with knee OA. To achieve the connecting effect by means of internet technology within the community setting, a specific software “FOR KOA” was purposely designed, which can be installed in the mobile phone and is capable of collecting the basic information of the participants recruited in this study, and monitoring the change of heart rate, blood pressure and oxyhemoglobin saturation from bracelet. The community-based interventions including and aerobics (power cycling and walking) and strength training. The hypothesis is that this collaborating strategy of the internet-based rehabilitation program and community-based interventions is feasible and efficacy to ameliorate the osteoarthritic pain and promote the physical function and quality of life for the patients with knee OA in the community setting.

## 2 Methods

### 2.1 Study design

**Figure 1:**
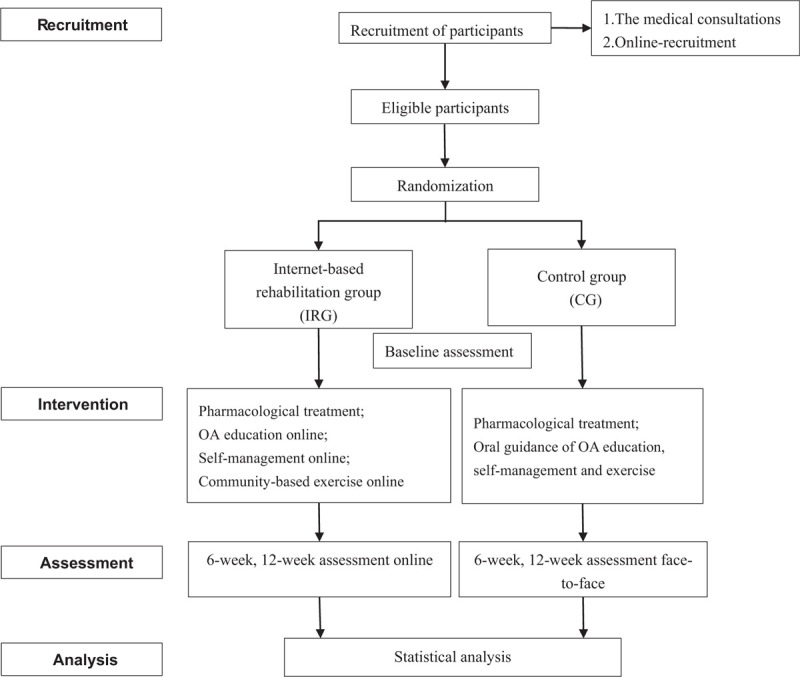
The flow chart of the trial.

The study is a 12-week, assessor-blinded randomized control trial, following the standard protocol: Recommendations for Interventional Trials (SPIRIT) guidelines.^[39]^ Meanwhile, the report will be presented as the CONSORT and TIDieR guidelines.^[39–42]^ The flow chart of the clinical trial is shown in Figure 1. Trial recruitment started on the 15^th^ June 2020. Completion of interventions and closure of the data set is planned for early spring of 2021.

### 2.2 Participant

#### 2.2.1 Inclusion criteria

Eligible participants will meet the following requirements: diagnosed with knee OA according to the 2018 Chinese Orthopedics Association (COA) guidelines for diagnosis and treatment of knee OA which satisfy following (1) and two criteria from (2) or (3) or (4) or (5):^[43]^ (1) recurrent knee pain in the last month; (2) narrowed joint space, subchondral cyst formation and bone sclerosis, or osteophytosis around joint margin on the radiographs in standing or load position in X-ray; (3) aged 50 years and over; (4) stiffness no more than 30 minutes in the morning; (5) palpable bone crepitation (fremitus) on movement of joint. In addition, participants will have the ability to use smart phones, communicate in Mandarin and understand Chinese characters.

#### 2.2.2 Exclusion criteria

Participants who meet any of the following criteria will not be able to participate in the trial: having other systemic rheumatic diseases that have been diagnosed at the knee; having fallen in nearly 6 months; ready for knee arthroplasty; all knee arthroplasty or other knee-related surgery were performed, and meniscal tear or anterior cruciate ligament (ACL) tear occurred in the past six months; there were other health problems that prevented them from participants were treated for knee OA in the past six months, including surgery, medication, and physical therapy; chronic kidney disease; cancer; dementia or severe cognitive impairment; mental illness; hospitalization for stroke, heart attack, heart failure, or surgery for arterial obstruction in the past three months; there were other health problems that prevented them from participating in the project; unable to sign informed consent. Table 1 lists the full eligibility criteria.

**Table 1:**
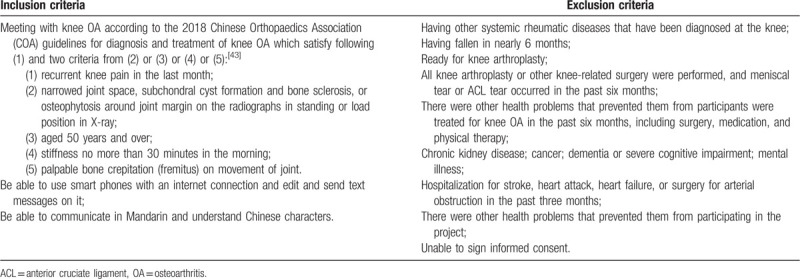
Trial inclusion/exclusion criteria.

#### 2.2.3 Recruitment

A multidisciplinary rehabilitative team has been built up, including the physicians, physiotherapists, nurses and bio-statistical professors. The potential participants will be recruited from the medical consultations in the 2 communities, supported by the department of rehabilitation medicine center of the Center of Rehabilitation Medicine, West China Hospital, Sichuan University. These communities had to previously signed the agreement with the West China Hospital of Sichuan University prior to study. The content will be explained to the patients from the community setting and they will be referred to a research staff responsible for collecting information for registration if they show interest in it. The research staffs are in charge of contacting the patients and making an appointment for medical evaluation. The eligible participants will be allocated into internet-based rehabilitation or control groups after the end of medical consultation. Furthermore, the recruitment can be performed through the Wechat app in the mobile to release the recruitment information. We will publish the details of the experiment in Wechat group, including the start time of the trial, the content to be carried out and the statement of relevant interests. People will fill out a spreadsheet to collect information if they are interested in this, and dedicated staffs will contact them for evaluation.

### 2.3 Interventions

#### 2.3.1 Internet-based rehabilitation group

**Figure 2:**
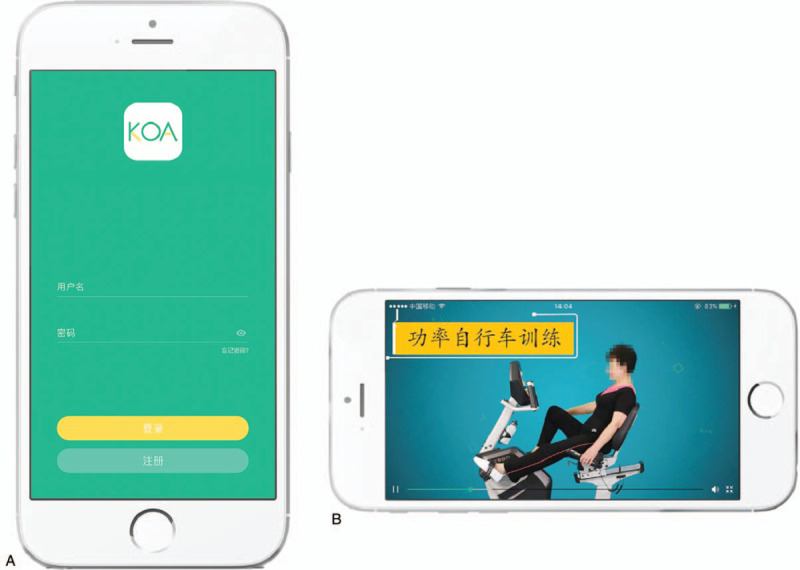
A The software “FOR KOA”: Login interface. B The software “FOR KOA”: Instruction video for cycle ergometer.

**Figure 3:**
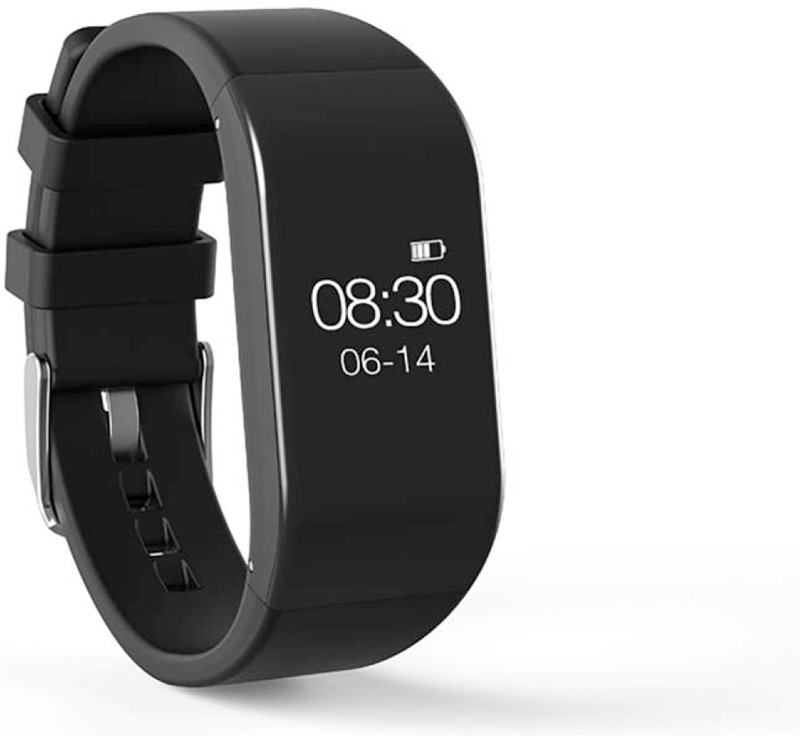
The bracelet for patients.

The purposely designed software “FOR KOA” enable the conduct of the internet-based rehabilitation programs, which mainly include arthritis education, self-management and land-based exercise programs according to the latest guidelines by Osteoarthritis Research Society International (OARSI).^[5]^ The bracelet matched with the software “FOR KOA” will automatically collect the heart rate, blood pressure and blood oxygen saturation of the participants during the exercise and display them, so as to allow the physical therapist to remotely monitor whether the participants’ exercise intensity meets the requirements (The software “FOR KOA”: A and B of Fig. 2; The bracelet for patients: Fig. 3). Participants will also be required to assess the outcome from the software “FOR KOA” at the beginning of the trial, after 6 weeks and 12 weeks. Participants may terminate the trial at any time and receive relevant treatment in case of symptom deterioration or other unexpected circumstances during the trial. During the trial, the celecoxib and glucosamine hydrochloride are also required.

##### 2.3.1.1 Osteoarthritis education

The purpose of osteoarthritis education is to let participants know their own situation, build up their confidence and encourage them to participate in treatment. Participants can communicate with physicians and physiotherapists through the application during this process. Physicians and physiotherapists can also push the content of knee OA on the application platform, including the introduction of popular science of knee OA, the latest research progress of knee OA rehabilitation, etc. Relevant contents will be published on the application platform every 2 weeks.

##### 2.3.1.2 Self-management

Self-management will guide participants in three aspects: emotion management, weight management and medication management. Emotional management will be guided by psychotherapists through voice and video via the application platform. The participants will upload the body mass index on the application every week, and read the weight management and diet nutrition information pushed by the application platform. Medication management will be conducted under the guidance of physicians. If participants take other drugs during the trial, they will also actively record them on the application platform.

##### 2.3.1.3 Land-based exercise programs

Land-based exercise programs include land aerobics (power cycling and walking) and strength training. Three times a week, 30 to 45 minutes each time, the exercise intensity was moderate (the range of metabolit equivalent is 3–6). The intensity of exercise is measured by the rating of perceived exertion (RPE). When the RPE reaches 12–14 or the heart rate reaches 70% of the maximum heart rate (maximum heart rate = 207-age ∗ 0.7), the moderate intensity of exercise is achieved.^[44]^ Participants will first assess under the remote guidance of physicians and physiotherapists in practice; then, physiotherapists will transmit exercise prescriptions and action teaching videos to participants through the application platform according to the assessment results; after physicians and physiotherapists confirming that they can correctly conduct action training through the feedback of participants’ image data, participants start to exercise program according to the exercise prescription. When the RPE of participants continues to decrease or the heart rate intensity fails to meet the requirements, the physiotherapist will make new exercise programs for them. The implementation place of exercise programs is in the community activity center and home. Power bikes will be placed in the activity center of community for every participant to do exercise.

The bracelet as wearable devices will be used to detect data of individuals in the process of exercise, monitor the movement of participants, and ensure the effective exercise of participants. The date of individuals in the process of movement will be uploaded every ten seconds, and all data will be saved in the database for storage. Physicians and physiotherapists who are responsible for guiding participants can log in from the computer and view the patient's movement data, which adjust the exercise prescription in real time. Furthermore, patients can leave messages to physicians and physiotherapists through the software platform instead of coming to the hospital in person for face-to-face communication. Physicians and therapists can also release relevant information of osteoarthritis education to patients through the software platform, and provide remote guidance to patients in all aspects.

#### 2.3.2 Control group

Oral guidance including arthritis education, self-management and land-based exercise will be delivered from physicians and physiotherapists after the first assessment to the participants. Based on the specific situation of participants, the appropriate adjustments of the oral guidance will be made and recorded in a datasheet for analysis. In addition, the participants will be allowed to receive further intervention once the symptoms worsen. During the trial, the celecoxib and glucosamine hydrochloride are also required.

### 2.4 Randomization and blinding

Participants will be randomly divided into the internet-based rehabilitation group and control group as the proportion of 1:1 according to the principle of random distribution after completing the baseline assessment for participants. The random numbers will be generated via computer software programs designed by statisticians who is blinded to the study content. The random number assigned to each group will be written on a piece of paper and placed in a sealed and opaque envelope. The envelope will be provided to the participants based on the randomization order. The assessors in the trial will not be aware of the random assignment for the participants. All the physicians and physiotherapists participating in the outcomes assessment and intervention guidance will receive a standardized training prior to this study, and will be allocated into these different two procedures during the trial. Physicians and physiotherapists involved in the assessment will not participate in the later experimental intervention. Data will be analyzed with blinding to group allocation in the data statistics.

### 2.5 Withdraw

The participants in both groups can withdraw from the trial during the study. The researchers will notify the withdraw ones of potential adverse events and related managements, and record the withdrawal information, such as the reason, time and contact person.

### 2.6 Outcome assessments

Outcomes assessments will be conducted at baseline, 6 weeks and 12 weeks post-intervention. All participants will be assessed by researchers who are blinded to the group assignment.

#### 2.6.1 Primary outcome measures

It is foremost to explore the feasibility of internet-based rehabilitation program for the patients with knee OA in the community setting. To assess feasibility, data completeness will be examined as the proportion of participants in the internet-based rehabilitation group who meet the inclusion criteria but are unwilling to participate in the trial, as well as the satisfaction at the end of trial. The compliance of the participants from internet-based rehabilitation group will also be used as a reference index for feasibility. Each time the participants exercise in the community setting they can sign in after starting the program as long as they wear wearable devices. We consider it as high compliance when the number of participants completing the exercise accounts for 90% or more of the required number of times.

The contents of the survey are the overall satisfaction of the participants with the use of the application platform, as well as the satisfaction with osteoarthritis education, self-management and land-based exercise program, as well as the satisfaction with wearable devices in the research process. The results of satisfaction survey were conducted by using 5-point Likert scale. Participants will choose one answer from “very satisfied”, “satisfied”, “general”, “dissatisfied” and “very dissatisfied” under each question. The answers above correspond to 5, 4, 3, 2, and 1 points respectively.^[45]^ The final total score is calculated. The higher the final score of the questionnaire, the higher the satisfaction of the participants.

#### 2.6.2 Secondary outcome measures

Pain of knee (s) will be assessed with a 11-point Numerical Rating Scale, 0 is no pain and 10 is worst possible pain.

The Western Ontario and McMaster Universities Osteoarthritis Index (WOMAC) will be used to measure pain, stiffness and physical function in the knee. WOMAC, a self-administered health-status instrument for patients with knee OA, consists of 24 items within three subscales: pain (five items), stiffness (two items) and physical function (17 items).^[46,47]^ All items are rated on a Likert scale of 0 (no symptoms)–4 (extreme symptoms), with a total range of 0–96 and higher scores indicating worse symptoms.

Quality of life will be assessed by means of the Short Form-36 quality-of-life questionnaire (SF-36). Zero points corresponds to the worst quality of life and 100 points corresponds to the best quality of life as put forward through the questionnaire.^[48]^

Adverse events will be recorded throughout the trial. The intensity and possible relationship with the treatment will be examined and described in detail if those once occurred.

Baseline assessment will be carried out after eligibility and informed consent have been confirmed. Demographic data referring to participant's age (in years), gender, height, weight, body mass index (BMI), physical functional data referring to physical activity (evaluated by the International Physical Activity Questionnaire (IPAQ) short version),^[49]^ education, occupation, source of income, hobbies and interests, marital, smoking and drinking status, medical history will all be collected.

### 2.7 Safety measurement

Adverse events of participants will be monitored during the intervention. Any adverse events, including exercise injuries, will be reported in detail by the study assistant of the study group using the adverse event case report form (CRF).

### 2.8 Sample size

According to the previous statistical study, the recommended sample size of the feasibility trial is at least 12 in each group.^[50]^ 20 participants are recruited in each group and a total of 40 participants are recruited allowing for not less than 20% dropout rate of the period. The participants will help us to evaluate the mean and variability of the test results in order to provide plate beads for future larger scale research.

### 2.9 Statistical analysis

To evaluate the feasibility of the study, we will calculate the proportion of enrolled participants out of all eligible patients, participants who dropout rates. Besides, a patient satisfaction survey for the use of the “FOR KOA” will be collected and analyzed. For the feasibility index, the method of statistical description is contingency table, composition ratio and statistical chart.

Means and standard deviations will be used to describe between-group statistics of 11-NRS, WOMAC and SF-36. The measurement data will be assessed for normal distribution using Shapiro-Wilk test for both groups. For the data description of quantitative index, using 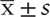 if it obeys normal distribution; if it does not obey normal distribution, use M ± (QU-QL). In statistical inference, repeated measurement analysis of variance (subject to normal distribution, homogeneity of variance and spherical symmetry), post-hoc test and rank sum test (not subject to homogeneity of normal distribution and variance) are used. Statistical analysis will be processed by SPSS software (version 24.0). The categorical variables will be compared by a chi-square test. *P* < 0.05 will be accepted as the threshold for statistical significance.

### 2.10 Data collection and management

The outcome and questionnaire data of IRG are stored on stored on secure servers Alibaba Cloud servers in China. The data of CG will be collected by CRF. Then, the CRF will be transcribed the into Alibaba Cloud servers by research assistants. Only the research team has access to the data. The details of data confidentiality and storage are included in the consent form and explained to participants by the research assistant during the baseline lab session. All information of the participants will be kept confidential and explained to them in the informed consent form. A formal data monitoring committee has not been created for this intervention; however, the project coordinator provides monthly reports on participant numbers and trial progress to the principal investigator. The final decision will be made by the principal investigator.

### 2.11 Patient and public involvement

Before the recruitment phase, we invited elderly people from communities to investigate the feasibility of design and interventions. According to their opinions, we avoided the time inconsistency. The burden of interventions included in this study will not be assessed by patients.

### 2.12 Ethics approval

The trial will be conducted in accordance with the Declaration of Helsinki. Ethics approval had been granted by the Clinical Trial and Biomedical Ethics Committee of the West China Hospital, Sichuan University (ethics reference: 2020 (457)). The study was registered in the Chinese Registry website (registered in ChiCTR. org with the identifier ChiCTR2000033397). Informed consent will be provided to all participants and signed prior to the trial. All study-related information of subjects will be protected during the trial.

### 2.13 Dissemination

The results of this study will be published at local, national or international rehabilitation conferences and submitted as manuscripts to peer-reviewed journals. The main findings of the study will also be shared with all participants and disseminated to researchers, health service providers, health care professionals and the public via the courses.

## 3 Discussion

This study is investigating whether an internet-based rehabilitation program combining wearable devices and software “FOR KOA” can be implemented in the community setting and improve physical function and pain for patients with knee OA at 12 weeks. If effective, the results of this study could contribute to a better understanding of the implementation of the internet-based rehabilitation program for patients with OA in the community setting.

Different from the previous study, we enroll the participants directly by holding medical consultations in the community setting. Compared with recruiting participants via print, radio, social media advertisements and the database of previous research projects, holding medical consultation in the community setting to enroll potential eligible patients can not only understand the living conditions of patients with knee OA, but also screen the early patients with OA and slow down the disease process by accepting rehabilitation in the community setting. In addition, one study suggested that the recruitment with medical professionals could result in more highly motivated samples with greater “readiness to change”.^[27]^

High adherence of treatment is necessary to improve the physical condition of patients,^[51]^ but the results were diverse in the previous studies with regard to internet-based rehabilitation.^[24,25]^ In the “Join2Move” program, 80% of participants completed the first one and 55% the second among the 9 modules, indicating the low adherence to internet-based rehabilitation.^[25]^ In contrast, the “PainCOACH” program showed high acceptability and adherence in that 91% of participants completed all 8 modules from 8 to 10 weeks.^[24]^ This difference between these two internet-based rehabilitation programs might be due to that the “Join2Move” program was fully automated without the mutual support from health care providers. In order to improve patients’ compliance in the internet-based rehabilitation, we have taken innovations in this study through employing appropriately the resource of community setting. At first, of the internet-based rehabilitation will be located mainly in the community setting rather than at home. The aerobic exercise and power bicycles, included in this internet-based rehabilitation programs, will increase the chances of communicating with their peers in the community setting, and thus promoting the willingness of participation through peer encouragement.^[31,52]^ Secondly, wearable devices will be used in the form of bracelets, offering the appropriate psychological hints to the participants and assisting the function data monitor and collection during the whole study. The beneficial effect of wearable technology on the improvement of clinical trial performance has been demonstrated in the previous studies.^[37,53,54]^

An important challenge in this study is for participants to learn how to use internet-based applications smoothly, although previous studies indicated that the elderly showed great interest in internet-based interventions.^[55,56]^ The software “FOR KOA” contained a system operation handbook and demonstration videos to enable the participants to comply with the requirements of internet-based rehabilitation programs. In addition, participants can also consult on how to use it via voice or video calls to the research team. If necessary, the research staffs will also provide a face-to-face consulting service. Participants’ caregivers will be included in the trial and encouraged to assist the elderly during the study.^[24]^

We hopefully build an internet-based rehabilitation model of knee OA in the Chinese community setting as well as to be constantly updated and upgraded the use of wearable devices and the software “FOR KOA” in the future so as to promote the development of community-based rehabilitation. A professional team that focuses on internet-based rehabilitation of knee OA in the community setting will also be established, improving the full cycle rehabilitation of patients with knee OA and promoting the use of community rehabilitation resources. The integration of clinical big data and improved wearable devices of knee OA rehabilitation will also be the best preparation for the implementation of the internet of things rehabilitation in the future.

## Author contributions

**Conceptualization:** Su-Hang Xie, Qian Wang, Cheng-Qi He.

**Formal analysis:** Li-Qiong Wang, Si-Yi Zhu, Yi Li.

**Funding acquisition:** Cheng-Qi He, Qian Wang.

**Project administration:** Cheng-Qi He.

**Writing – original draft:** Su-Hang Xie.

**Writing – review & editing:** Qian Wang, Yi Li, Cheng-Qi He.
